# Risk Factors Associated With Diabetic Retinopathy: A Cross-Sectional Study Within Palestinian Patients in Northern West Bank

**DOI:** 10.3389/fcdhc.2021.736715

**Published:** 2021-10-12

**Authors:** Johnny Amer, Raghad Suboh, Manar Abualrob, Amira Shaheen, Abdul Raheem Abu Shanab

**Affiliations:** ^1^ Physiology, Pharmacology & Toxicology Division, An-Najah National University, Nablus, Palestine; ^2^ Division of Public Health, Department of Biomedical Sciences, An-Najah National University, Nablus, Palestine; ^3^ Department of Applied and Allied Medical Sciences, Faculty of Medicine and Health Sciences, An-Najah National University, Nablus, Palestine

**Keywords:** diabetic retinopathy, diabetes mellitus, northern West Bank, duration of DM, prevalence

## Abstract

Risk factors associated with diabetes mellitus (DM) have been widely researched worldwide, but the determinants of these factors among diabetic retinopathy (DR) in Palestine are currently unclear. We aimed to assess the prevalence of DR among DM in Northern West Bank and identify factors associated with DR natural history. Patients with Type 2 diabetes (T2D) (n = 300, age > 18 years) from a main diabetic center covering all northern provinces of Palestine were enrolled to this cross-sectional research. Demographic information including age, sex, and duration of T2D was obtained. Moreover, HbA1C, BMI, hypertension (HTN), controlled T2D, current smoking, and total cholesterol level were assessed. Potential correlations between these factors and DR diagnosed by ophthalmologist were evaluated using different tests on SPSS version 22. Prevalence of DR among our population was 30%; 47.8% of these patients showed mild non-proliferative DR (NPDR), 23.3% moderate NPDR, 16.7% severe NPDR, and 12.2% proliferative DR (PDR). Univariate logistic regression analysis showed age (p = 0.007), HTN (p = 0.022), uncontrolled T2D (p = 0.025), and duration of T2D (<0.001) were mostly associated with DR while multivariate logistic regression showed duration of T2D as the major and solely risk factor for prevalence of DR (p < 0.0001) and were positively correlated with severities of NPDR and being a strong predictor in the PDR (p = 0.001). We identified several important risk factors that affect DR, which could assist to develop effective strategies for metabolic disease prevention among populations in Palestine. Furthermore, our data suggest a necessity to control sugar serum levels and HTN.

## Introduction

Diabetes mellitus (DM) is one of the most common chronic diseases worldwide, and it continues to increase in prevalence and disease burden (1). Diabetic retinopathy (DR), a common complication of DM, is the leading cause of impaired vision in adults worldwide (2). Patients with DR may suffer from damaged blood vessels of the light-sensitive tissue at the back of the eye (retina) and any form of diabetes, type 1, type 2, or gestational may lead to DR complications. Hypertension, smoking, hyperlipidemia, and some races were suggested as factors for DR progressions among DM in patients (1, 2).

DR is largely asymptomatic in the early stages, and there is a need for regular eye screening for patients with diabetes to enable timely diagnosis and subsequent management of the condition (3). Although in practice the early clinical features of DR complication are evident in the fundus on ophthalmoscopic examination, the initial diagnosis of diabetic retinopathy may be based on functional changes in electroretinography (ERG), retinal blood flow, and retinal blood vessel calibre (4). Thus, DR is currently categorized based on the presence of vascular (and closely associated) lesions and on the other hand through the absence or presence of neovascularization. There are two general categories: (a) non-proliferative diabetic retinopathy (NPDR) and (b) proliferative diabetic retinopathy (PDR) (2). Several studies recently have focused on the prognostic issues of DR; however, there is a significant lack of awareness of this disease in terms of risk factors and its outcomes. We thus conducted a population-based survey among Palestinian population with Type 2 diabetes (T2D). The purpose of this study the prevalence and identify potential risk factors of DR among T2D patients.

## Patients and Methods

### Study Design and Study Population

This study is a cross-sectional study of T2D patients (n = 300, age above 18 years) based on current World Health Organization diagnostic criteria for diabetes (5). The study on patients with T2D recruitment was conducted from February to October 2020 from the Palestine Diabetes National Center Nablus. The center covers patients from all northern geographical provinces of the West Bank, Palestine, including Nablus, Jenin, Tulkarm, Tubas, and Salfit. The patients were randomly selected from patient data profiles as the first 150 male and the first 150 females from the files that were arranged alphabetically with a total number of 300 patients who underwent fundoscopic examination and have regular follow-up visits to the institute since 2016 until now. Patients who take Avastin and had laser therapy were excluded from the study. The ethics committee of An-Najah National University approved the study, and written informed consent was obtained from all participants during recruitment. All methods were performed in accordance with the relevant guidelines and regulations.

### Information Evaluated in This Study

Detailed information was collected with regard to sex, age (>18), cigarette smoking, alcohol use, and residency. In addition to blood pressure measurement, physical examinations included body height, weight, and circumferences of the waist, hip, and abdomen. Moreover, hemoglobin A1C (HbA1C) and total cholesterol levels were included.

### Diabetic Retinopathy Diagnosis

Diabetic retinopathy was diagnosed by funduscopy. In cases where diabetic ophthalmic complications were suspected, then additional imaging modalities were used, including optical coherence tomography for the diagnosis of diabetic macular edema, and fluorescein angiography in cases where neovascular complications like optic disk or retinal neovascularization (i.e., proliferative diabetic retinopathy) were suspected.

### Definitions

Hypertension was defined as an average systolic blood pressure (SBP) of ≥140 mmHg and/or an average diastolic blood pressure (DBP) of ≥90 mmHg, or current use of any medication for treating hypertension within 2 weeks or any combination of the above. (6 Currently smoking patients were defined as active smokers for the last 3 years prior and during the data collection. An HbA1c above 7% was identified as uncontrolled T2D. A normal level of total cholesterol in blood is defined as total blood cholesterol below 5.18 mmol/l. Body mass index (BMI) was calculated as the ratio of weight to height squared (kg/m^2^). According to standard criteria in Palestinian adults, participants with a BMI ≥24 and <28 kg/m^2^ were classified as overweight, and those with a BMI ≥28 kg/m^2^ were classified as obese (5).

### Statistical Analysis

Continuous variables are presented as means [standard deviations (SD)], and categorical variables are expressed as percentages [95% confidence intervals (Cis)]. Categorical variables were analyzed using the chi-squared test. The risk factors of BMI, age, smoking, total cholesterol, and uncontrolled T2D were analyzed by bivariate analysis. Determinants of duration of T2D BMI, currently smoking, hypertension (HTN), total cholesterol, and uncontrolled T2D were assessed by multivariate linear regression analysis after adjustment for confounding factors that were statistically significant. Statistical significance was defined as a two-tailed p < 0.05. Statistical analyses were undertaken using the computer software SPSS (version 15.0 for Windows; SPSS, Chicago, IL, USA).

## Results

### Descriptive Characteristics of Patients

The mean age of T2D patients was 58.82 ± 10.3 years, the mean duration of T2D was 8.9 ± 7.3 years, and the mean BMI was 30.28 ± 5.4; 133 (34.4%) of patients were smokers, 197 patients (65.4%) have HTN, and 130 patients (34.3%) have high total cholesterol.

A total of 300 patients were included in this study, with equal numbers of males and females. The prevalence of DR was 30% (90 patients), distributed as 43 patients with mild NPDR (14.3%), 21 patients with moderate NPDR (7.0%), 15 patients with severe NPDR (5.0%), and 11 patients with PDR (3.7%). DR occurred in 49 males and 41 females with a 1.2:1 male to female ratio.

Our results summarized in **Table 1** revealed no significant association between BMI, currently smoking patients, and gender with DR. On the other hand, age, duration of T2D, HTN, uncontrolled T2D, and cholesterol were found significantly associated with the occurrence of DR with p-values 0.007, <0.001, 0.021, 0.020, and 0.003, respectively.

**Table 1:** The association between risk factors and DR.

	With DR n = 90	Without DR n = 210	
Age ± SD (years) min–max	61 ± 8 (39–78)	58 ± 11 (23–88)	t = 2.735 *p* = 0.007
BMI ± SD (kg\m^2^) Min–max	30.44 ± 5.5 (22–52.5)	30.22 ± 5.42 (16.5–52)	t = 0.33 *p* = 0.742
Duration of T2D ± SD (years) min–max	13.3 ± 7.2 (2–35)	7 ± 6.5 (0–28)	t = 7.540 *p* < 0.001
Currently smoking	Yes n = 38 (42.2%) No n = 52 (57.8%)	Yes n = 95 (45.2%) No n =115 (54.8%)	X^2^ = 0.232 *p* = 0.630
HTN ***^a^***	Yes n = 68 (75.6%) No n = 22 (24.4%)	Yes n = 129 (61.7%) No n = 80 (38.3%)	X^2^ = 5.356 *p* = 0.021
Uncontrolled T2D (HbA1C > 7%)	Yes n = 84 (93.3%) No n = 6 (6.7%)	Yes n = 174 (83.3%) No n = 35 (16.7%)	X^2^ = 5.402 *p* = 0.020
Cholesterol (normal is below 5.18 mmol/l)	Abnormal = 27 (31.4%) Normal = 59 (68.6%)	Abnormal = 103 (50.2%) Normal = 102 (49.8%)	X^2^ = 8.708 *p* = 0.003
Gender	Female = 41 (45.6%) Male = 49 (51.9%)	Female = 109 (51.9%) Male = 101 (54.4%)	X^2^ = 0.773 *p* = 0.379

^a^Average systolic blood pressure (SBP) of ≥140 mmHg and/or an average diastolic blood pressure (DBP) of ≥90 mmHg.

### Logistic Regression Model for Factors Predicting DR

**Figure 1:**
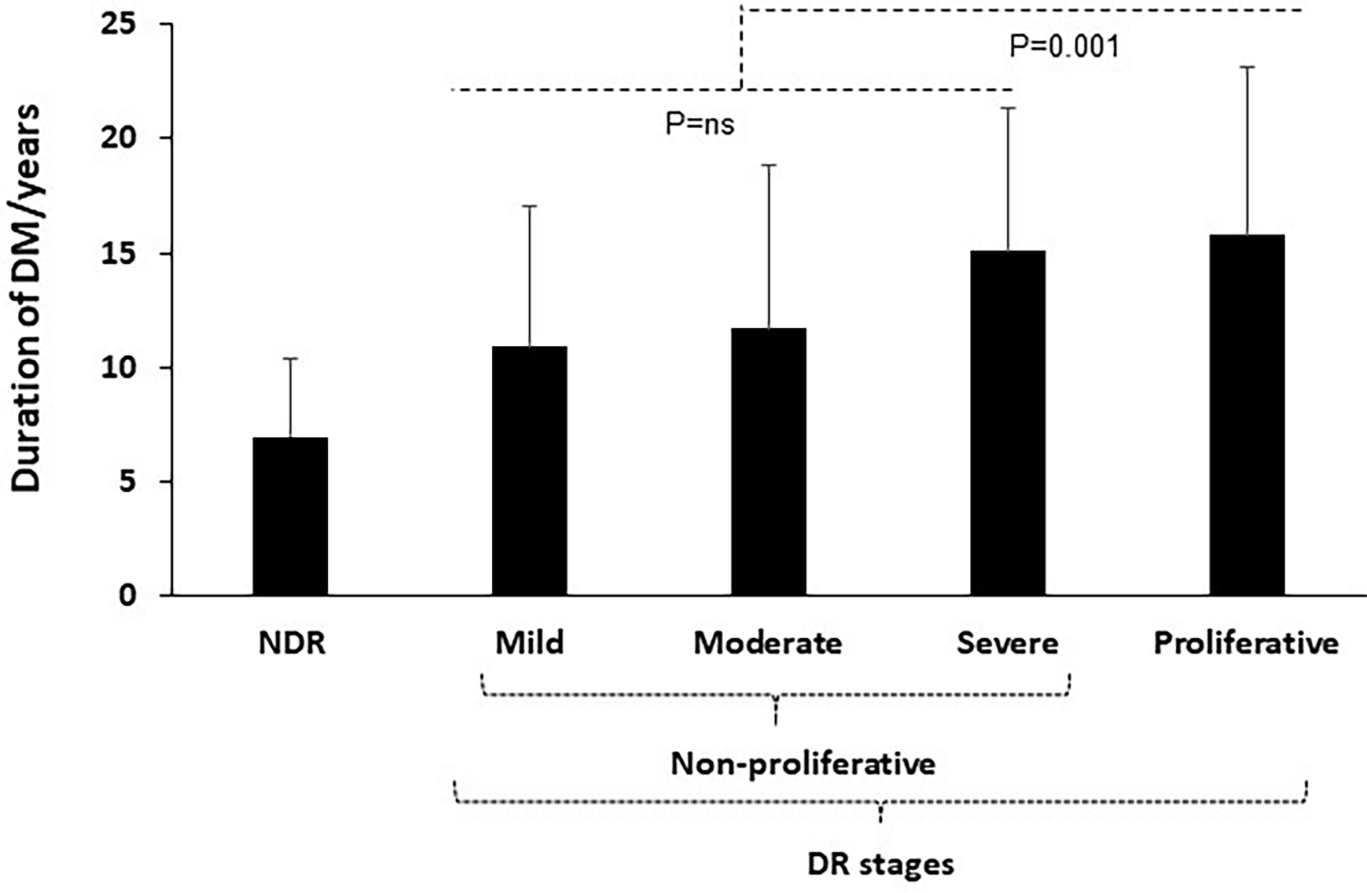
The correlation between duration of T2D/years and severties of DR.

The factors predicting DR are summarized in **Table 2**. The table displays the univariate regression of DR with other variables indicating the association between DR and duration of T2D, older age, presence of HTN, currently smoking patients, uncontrolled T2D (which is defined as HbA1c more than 7%), and the presence of normal level of total cholesterol in blood (defined as total blood cholesterol less than 200 mg/dl). Moreover, the multiple regression analysis in **Table 3** shows a highly significant positive association of only duration of T2D with DR (p < 0.001) while no association between DR and HTN, currently smoking, uncontrolled T2D, age, and total cholesterol was noticed in contrary to the findings in the univariate regression. **Figure 1** shows a linear correlation of duration of T2D/years with the severities of DR and being a strong predictor in the PDR (p = 0.001).

**Table 2:** Univariate logistic regression analysis model.

	**B**	**SE**	**Wald**	***p* value**	**Odds**	**95% CI for OR**	
						Lower	Upper
BMI (kg\m^2^)	0.008	0.023	0.109	0.741	1.008	0.963	1.055
Duration of T2D (years)	0.122	0.019	40.554	<0.001	1.130	1.088	1.173
Age (years)	0.035	0.013	7.166	0.007	1.036	1.009	1.062
Currently smoking	-0.123	0.254	0.232	0.630	0.885	0.537	1.357
HTN ***^a^***	0.651	0.284	5.265	0.022	1.917	1.100	3.342
Uncontrolled T2D (HbA1C > 7%)	1.035	0.461	5.035	0.025	2.816	1.140	6.957
Normal cholesterol (normal is below 5.18 mmol/L)	-0.791	0.271	8.523	0.004	0.453	0.266	0.771

^a^Average systolic blood pressure (SBP) of ≥140 mmHg and/or an average diastolic blood pressure (DBP) of ≥90 mmHg.

**Table 3:** Multivariate logistic regression analysis model.

	**B**	**SE**	**Wald**	***p* value**	**Odds**	**95% CI for OR**	
						Lower	Upper
Age (years)	0.004	0.016	0.051	0.822	1.004	0.973	1.035
Duration of T2D (years)	0.112	0.021	27.303	0.000	1.118	1.072	1.166
BMI (kg\m^2^)	0.010	0.028	0.133	0.716	1.010	0.956	1.068
Currently smoking	0.064	0.304	0.044	0.834	1.066	0.587	1.934
Uncontrolled T2D (HbA1C > 7%)	0.808	0.544	2.205	0.138	2.244	0.772	6.524
Cholesterol (normal is below 5.18 mmol/l)	-0.385	0.305	1.595	0.207	0.680	0.374	1.237
HTN	0.574	0.325	3.121	0.077	1.775	0.939	3.356
Constant	-4.549	1.920	5.616	0.018	0.011		

## Discussion

This is the first study to report factors influencing DR severities among T2D patients aged ≥18 years in northern Palestine. To date, there are a limited number of studies published on retinopathy in patients with T2D in Palestine. Therefore, this study aimed to assess the prevalence of DR among Palestinian T2D patients and highlight the risk factors associated for its prediction. In our current study, the risk factors associated with DR of HbA1c and HTN were associated with severe DR (6–10). Tight glycemic and blood pressure control remains the cornerstone in the primary prevention of DR (11). However, glycemic control was considered as the most important factor for preventing retinopathy in patients with diabetes (12) and intensive therapy effectively delays the onset and slows the progression of diabetic retinopathy (12). Our findings appear to corroborate the results reported in other studies indicating HTN and glycemic control appear to have a direct relationship with development of DR.

In the duration of diabetes, a significant association was noticed between longer duration of T2D and DR occurrence. Similar findings were found in several other studies (6, 7, 10, 13, 14), which considered it as the predictor of DR (15). Moreover, in line with other studies (12, 13, 16), there was no significant association indicated between gender and the occurrence of DR. In addition, our results indicated no significant association of DR with gender, unlike other studies (12, 14) that showed an increase in the incidence of DR in male patients. Regarding BMI, studies showed controversial data; some show no association with the development of DR (13, 17), while others showed increased BMI with a positive association with DR (3, 18, 19). Our findings demonstrated BMI as a non-significant indicator associated with risks of DR. Majority of studies show that smoking is not likely to be an important risk factor for DR (12, 17, 20, 21). Our results were consistent with these studies. However, a few studies demonstrate a relationship between smoking and DR (5). DR incidences were reported in elder patients (14), and age appeared as an independent risk factor for DR (22). Our findings support this evidence, as do other studies (12, 13).

A controversial study displayed an association between cholesterol and DR. A previous study demonstrated that reducing elevated blood lipids could have an impact to slow the progression of retinopathy (23). However, high levels of total cholesterol had been reported to be associated with lower risk of DR, and it could be a protective factor against DR (24). In the current report, patients with DR [59 (68.6%)] had normal serum levels of total cholesterol as compared to patients without DR [102 (49.8%)] (χ^2^ = 8.708 and *p* = 0.003). A study performed in Gaza Strip among male patients with T2D demonstrated a significantly higher prevalence of neuropathy, nephropathy, cardiovascular disease, and recurrent infections among patients with DR as compared to those without DR (25).

In our sample, we introduced a new data of a cross-sectional study indicating 30% prevalence of DR in northern West Bank, Palestine. DR stage’s severities showed approximately an equal distribution between males and females (1.2:1 male-to-female ratio). Although sample size could be a limited factor for the sampling population, following the reduced impact of confounding variables, duration of T2D could be considered as the most significant risk factor for DR and may be linearly correlated with DR severities.

## Data Availability Statement

The original contributions presented in the study are included in the article/supplementary material. Further inquiries can be directed to the corresponding author.

## Ethics Statement

The ethics committee of An-Najah National University approved the study. The patients/participants provided their written informed consent to participate in this study. Written informed consent was obtained from the individual(s) for the publication of any potentially identifiable images or data included in this article.

## Author Contributions

JA formulated the research question, analyzed the data, and had primary responsibility for the final content. RS and MA carried out the research and wrote the draft. AS checked out the statistical analysis. AA revised the clinical data. All authors contributed to the article and approved the submitted version.

## Conflict of Interest

The authors declare that the research was conducted in the absence of any commercial or financial relationships that could be construed as a potential conflict of interest.

## Publisher’s Note

All claims expressed in this article are solely those of the authors and do not necessarily represent those of their affiliated organizations, or those of the publisher, the editors and the reviewers. Any product that may be evaluated in this article, or claim that may be made by its manufacturer, is not guaranteed or endorsed by the publisher.

## Abbreviations

T2D, type 2 diabetes; DR, diabetic retinopathy DR; HTN, hypertension; NPDR, non-proliferative diabetic retinopathy; PDR, proliferative diabetic retinopathy.
